# Non-contrast MR angiography at 1.5 Tesla for aortic monitoring in Marfan patients after aortic root surgery

**DOI:** 10.1186/s12968-017-0394-y

**Published:** 2017-10-30

**Authors:** Simon Veldhoen, Cyrus Behzadi, Alexander Lenz, Frank Oliver Henes, Meike Rybczynski, Yskert von Kodolitsch, Thorsten Alexander Bley, Gerhard Adam, Peter Bannas

**Affiliations:** 1 Department of Diagnostic and Interventional Radiology and Nuclear Medicine, University Medical Center Hamburg-Eppendorf, Hamburg, Germany; 2 Department of Diagnostic and Interventional Radiology, University Hospital Würzburg, Bavaria, Germany; 3 Department of General and Interventional Cardiology, University Medical Center Hamburg-Eppendorf, Hamburg, Germany

## Abstract

**Background:**

Contrast-enhanced cardiovascular magnetic resonance angiography (CE-CMRA) is the established imaging modality for patients with Marfan syndrome requiring life-long annual aortic imaging before and after aortic root replacement. Contrast-free CMRA techniques avoiding side-effects of contrast media are highly desirable for serial imaging but have not been evaluated in the postoperative setup of Marfan patients. The purpose of this study was to assess the feasibility of non-contrast balanced steady-state free precession (bSSFP) magnetic resonance imaging for aortic monitoring of postoperative patients with Marfan syndrome.

**Methods:**

Sixty-four adult Marfan patients after aortic root replacement were prospectively included. Fourteen patients (22%) had a residual aortic dissection after surgical treatment of type A dissection. bSSFP imaging and CE-CMRA were performed at 1.5 Tesla. Two radiologists evaluated the images regarding image quality (1 = poor, 4 = excellent), artifacts (1 = severe, 4 = none) and aortic pathologies. Readers measured the aortic diameters at defined levels in both techniques. Statistics included observer agreement for image scoring and diameter measurements and ROC analyses for comparison of the diagnostic performance of bSSFP and CE-CMRA.

**Results:**

Both readers observed no significant differences in image quality between bSSFP and CE-CMRA and found a median image quality score of 4 for both techniques (all *p* > .05). No significant differences were found regarding the frequency of image artifacts in both sequences (all *p* > .05). Sensitivity and specificity for detection of aortic dissections was 100% for both readers and techniques. Compared to bSSFP imaging, CE-CMRA resulted in higher diameters (mean bias, 0.9 mm; *p* < .05). The inter-observer biases of diameter measurements were not significantly different (all *p* > .05), except for the distal graft anastomosis (*p* = .001). Using both techniques, the readers correctly identified a graft suture dehiscence with aneurysm formation requiring surgery.

**Conclusion:**

Unenhanced bSSFP CMR imaging allows for riskless aortic monitoring with high diagnostic accuracy in Marfan patients after aortic root surgery.

## Background

Marfan syndrome is a genetic disorder of the connective tissue with autosomal dominant inheritance. Its prevalence has been indicated by one in 5.000–10.000 individuals [1, 2]. Mutations in the FBN1 gene encoding the protein Fibrillin cause general connective tissue insufficiency [3, 4]. Progressive dilation of the aortic root at the sinuses of Valsalva is the most common cardiovascular complication of Marfan syndrome [5, 6]. Aortic root aneurysms may cause aortic dissection and represent the main cause of death in undetected Marfan syndrome [5–7]. Nowadays, pharmacotherapies reduce the progression rate of aortic dilation, and elective repair of the aortic root has significantly improved the survival of Marfan patients [8, 9]. However, life-long annual aortic imaging is mandatory for Marfan patients to determine if and when aortic root replacement is indicated [8, 10].

Cardiovascular magnetic resonance angiography (CMRA) has been established for serial monitoring of aortic pathologies [11, 12]. Contrast-enhanced CMRA (CE-CMRA) is considered the reference technique in CMRA imaging [11, 12]. However, gadolinium based contrast agents bear the risk of side effects such as hypersensitivity, nephrogenic systemic fibrosis or cerebral gadolinium deposition [13, 14]. This highlights the need for non-contrast CMRA techniques for life-long annual imaging of Marfan patients. Several non-contrast CMRA techniques have been evaluated for imaging the aorta [15, 16]. Among these, balanced steady-state with free precession (bSSFP) sequences offer inherent high contrast between blood and background tissues, thereby allowing for optimal delineation of the aortic wall [15, 17, 18]. Recent studies confirmed that bSSFP sequences allow for accurate pre-operative monitoring of aortic root diameters in Marfan patients [19–21].

Prolonged survival after aortic surgery has led to an increase of Marfan patients with aortic complications beyond the root [22]. Elective replacement of the aortic root removes the most important predilection site for aneurysms, but the distal aorta remains at risk for dilation [9, 23]. Complications such as aneurysms and dissections in the distal aorta are doubled in Marfan patients with previous elective aortic surgery [9]. However, even after aortic root replacement the ascending aorta remains in focus of interest as life-threatening complications such as suture dehiscence with development of aneurysm in the graft region may occur [24, 25]. Thus, life-long annual aortic imaging is mandatory before and after aortic root replacement for early detection of proximal as well as distal aortic complications [6, 8, 9, 26–28].

Thorough evaluation of non-contrast bSSFP sequences for imaging in the post-operative set up of Marfan has not yet been performed. However, minimizing the risks of contrast media associated side-effects is highly desirable in Marfan patients undergoing lifelong annual aortic imaging also after aortic surgery. Therefore, we aimed to assess the feasibility of non-contrast bSSFP imaging for monitoring of aortic diameters and dissections in Marfan patients after aortic root replacement.

## Methods

### Study population

The prospective cohort study was approved by the institutional review board. All patients provided written informed consent. We included 64 adult patients (42 men; 22 women; age range 19–73 years; mean age 44 ± 13 years) with confirmed Marfan after aortic root surgery. Forty-six patients (72%; 30 men; 16 women; age range 19–73 years; mean age 44 ± 13 years) underwent prophylactic aortic root surgery due to increased aortic root diameters. Eighteen patients (28%; 12 men; 6 women; age range 34–72 years; mean age 47 ± 9 years) underwent aortic root replacement due to acute type A dissection. At the time of CMR imaging, 50 patients (78%) had no dissection, while 14 patients (22%) had a known residual aortic dissection after surgical treatment of type A dissection. These dissections were confirmed by previous cross sectional imaging examinations and/or known from surgical reports. Eleven patients (17%) underwent additional aortic surgery distally to the aortic root: Five patients had aortic arch replacement, two had prosthesis of the descending aorta, and four had thoracic endovascular aortic repair of the descending aorta.

All included Marfan patients underwent CMR imaging as part of their routine postoperative follow-up. The mean interval between aortic root surgery and CMR study was 6.9 ± 5.9 years. Minors and patients with contraindications for CMR were not included. The local Universitary Marfan Center associated with the University Heart Center established the Marfan diagnosis in each subject based on evaluation according to the latest Ghent nosology as well as genetic analyses with sequencing of the FBN1 gene [2, 11, 29].

### CMR imaging

CMR imaging was performed using a 1.5 Tesla scanner equipped with a five-channel coil for cardiac imaging (Achieva, Philips Medical Systems, The Netherlands). Electrocardiography (ECG)-leads were placed in typical manner for cardiac triggering.

ECG-gated non-contrast 2D bSSFP imaging with sensitivity encoding (SENSE) was triggered to the end-diastolic phase of the cardiac cycle for minimization of motion artefacts and acquired during end-expiratory breath-hold as recommended by current guidelines [11]. Images were acquired in the transversal and coronal plane as well as in para-sagittal orientation aligned with the curvature of the aortic arch during a single breath-hold for each orientation. [19, 21]. Image parameters were as follows: TR/TE, 3.2/1.6 ms; flip angle, 90°; field of view, 430 mm × 302 mm; matrix, 256 × 180; number of slices, 20; in-plane resolution, 1.7 mm × 1.7 mm; slice thickness, 10 mm; SENSE-factor, 2; acquisition time for each stack, 12–16 s (depending on the individual heart rate).

Contrast-enhanced 3D CMRA of the entire aorta was performed after automatic injection (2 ml/s) of gadopentetate dimeglumine (Gd-DTPA, Magnevist, Bayer-Schering Pharma AG, Germany) at a dose of 0.1 mmol/kg bodyweight into an antecubital vein. Scanning parameters of the gradient-echo T1-weighted sequence were as follows: TR/TE, 4.8/1.4 ms; flip angle, 40°; field of view, 450x360x90–130 mm; matrix, 368x189x25–36. True spatial resolution was 1.2 × 1.9 × 3.6 mm^3^, interpolated to 0.9 × 0.7 × 3.6 mm (512 × 512 matrix). To determine the scan delay after contrast injection a 2 ml test bolus was used. Imaging was started at the time of contrast arrival in the descending aorta during end-expiratory breath hold. Two separate post-contrast datasets were acquired with a 10-s respiration interval. The series with superior illustration of the contrast bolus was picked for image analyses. Total acquisition time, including the test bolus, ranged from 90s to 120 s depending on the patient anatomy and the field of view.

### Qualitative image analyses

Two radiologists ((S.V and C.B.) with five and four years of experience in cardiovascular imaging, respectively) performed individual reading of anonymized and randomized images acquired with bSSFP and CE-CMRA sequences. Readers evaluated entire series of all imaging planes.

First, the overall subjective image quality of the aorta was rated based on a four-point scale: Score of 1 = poor image quality, poorly defined anatomic details, poor diagnostic confidence; score of 2 = reduced image quality, limitations in anatomic detail, impairment of diagnostic confidence; score of 3 = good image quality, clear anatomic details, no impairment of diagnostic confidence; score of 4 = excellent image quality, distinct anatomic details, full diagnostic confidence [30].

Second, the presence of image artifacts at the site of the aortic root graft was scored on a four-point scale: Score of 1 = severe artifacts; score of 2 = moderate artifacts; score of 3 = minor artifacts; score of 4 = no artifacts [31].

Third, the presence of aortic dissection was scored on a five-point scale (score of 1 = certainly dissection, full diagnostic confidence; score of 2 = probably dissection, impairment of diagnostic confidence; score of 3 = unsure; score of 4 = probably no dissection, impairment of diagnostic confidence; score of 5 = certainly no dissection, full diagnostic confidence) [31, 32].

Fourth, the readers were asked to note the presence of any other relevant aortic pathology such as aneurysms.

### Quantitative image analyses

**Fig. 1:**
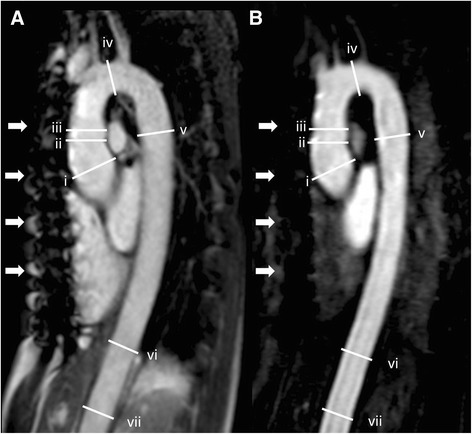
Aortic diameter measurements: Parasagittal (**a**) non-contrast 2D bSSFP and (**b**) 3D CE-CMRA of a 37-year-old woman with Marfan syndrome 5 years after valve-sparing aortic root replacement (David procedure). White lines indicate the seven measurement levels along the aorta. From proximal to distal: i) middle of the aortic root graft, ii) distal anastomosis of the graft, iii) ascending aorta at the level of the pulmonary trunk, iv) mid aortic arch, v) descending aorta at the level of the pulmonary trunk, vi) aorta at the level of the diaphragm, and vii) aorta proximal to the celiac trunk. Both readers rated the image quality and artifact level as 4 points (best quality and no diagnosis interfering artifacts, respectively) for both bSSFP and CE-CMRA. However, note the presence of artifacts caused by sternal cerclages in both examinations (white arrows)

Both readers performed aortic diameter measurements on identically orientated para-sagittal source images of non-contrast bSSFP and CE-CMRA sequences. Aortic diameters were measured perpendicular to the blood-filled lumen [21]. Readers were free to choose appropriate slices displaying the maximal profile of the aorta from the stacks of para-sagittal images [21]. The following measuring points were determined: i) middle of the aortic root graft, ii) distal anastomosis of the graft, iii) ascending aorta at the level of the pulmonary trunk, iv) mid aortic arch between the branching of the left carotid and the left subclavian artery, v) descending aorta at the level of the pulmonary trunk, vi) aorta at the level of the diaphragm, and vii) aorta proximal to the coeliac trunk **(**Fig. 1 **)** [6, 11]. Defined aortic measuring levels that were replaced by implanted grafts were skipped and excluded from further analyses. Diameters were measured three times in each image series: Reader 1 performed two measurements with an interval of 6 weeks for assessment of the intra-observer agreement. Reader 2 performed a third measurement for assessment of the inter-observer agreement.

### Statistical analysis

Wilcoxon signed-rank test was used to assess differences in the subjective scoring of the image quality and the degree of artifacts in bSSFP imaging and CE-CMRA. Intraclass correlation coefficient (ICC) was calculated to assess inter-observer agreement regarding these subjective scorings. Sensitivity and specificity regarding detection of aortic dissection in bSSFP imaging and CE-CMRA was assessed using receiver operating characteristic (ROC) analyses. Scorings were clustered in identified dissections with high levels of confidence (scores of 4 and 5) vs. no dissection (scores 1–3). The resulting areas under the curve (AUC) with corresponding 95% confidence intervals (CI) were used to compare the performance of the techniques regarding detection of aortic dissections.

Bland–Altman and ICC analyses were used to assess intra- and inter-observer agreement regarding diameter measurements obtained from bSSFP imaging and CE-CMRA. A paired t-test was performed for comparison of mean differences and F-test for comparison of variances. Pearson’s correlation was calculated to determine the correlation between diameters assessed by bSSFP and CE-CMRA. *P*-values <.05 were considered as statistically significant. Statistical analyses were performed using SPSS v. 20.0 (International Business Machines, Inc., Chicago, Illinois, USA) and Excel v. 15.26 (Microsoft, Redmond, Washington, USA).

## Results

All CMR studies were performed without periprocedural complications and all examinations were included into the evaluation.

### Overall aortic image quality

All examinations were diagnostic. None of the CMR examinations resulted in poor diagnostic confidence (score 1) regarding the overall image quality. Reader 1 and reader 2 found good to excellent image quality in 97 and 95% of the bSSFP examinations and in 98% of CE-CMRA scans, respectively. There was no significant difference in image quality scores between SSFP imaging and CE-CMRA (reader 1, *p* = .37; reader 2, *p* = .16). The median image quality score was 4 for both readers and techniques. The inter-rater agreement was comparable (ICC = 0.63 for bSSFP vs. 0.56 for CE-CMRA). Detailed results of image quality ratings are provided in Table 1.

**Table 1:** Results of the qualitative measurements

Qualitative Reading and Technique	Reader 1		Reader 2		ICC
Overall aortic image quality					
2D bSSFP	n	%	n	%	0.63 [0.45–0.76]
Score 1 – Poor	0	0.0	0	0.0	
Score 2 – Reduced	2	3.1	3	4.7	
Score 3 – Good	11	17.2	20	31.3	
Score 4 – Excellent	51	79.7	41	64.1	
Median score	4		4		
3D CE-CMRA	N	%	n	%	0.56 [0.37–0.71]
Score 1 – Poor	0	0.0	0	0.0	
Score 2 – Reduced	1	1.6	1	1.6	
Score 3 – Good	10	15.6	16	25.0	
Score 4 – Excellent	53	82.8	47	73.4	
Median score	4		4		
P (Wilcoxon)	0.37		0.16		
Artifact scoring at the aortic root					
2D bSSFP	n	%	n	%	0.81 [0.62–0.90]
Score 1 - Severe	2	3.1	2	3.1	
Score 2 - Moderate	4	6.3	6	9.4	
Score 3 - Minor	9	14.1	20	31.3	
Score 4 - Absent	49	76.6	36	56.3	
Median score	4		4		
3D CE-CMRA	n	%	n	%	
Score 1 - Severe	0	0.0	0	0.0	0.65 [0.46–0.78]
Score 2 - Moderate	0	0.0	7	10.9	
Score 3 - Minor	14	21.9	11	17.2	
Score 4 - Absent	50	78.1	46	71.9	
Median score	4		4		
P (Wilcoxon)	0.18		0.12		

Categorial rating of non-contrast 2D–balanced steady state free precession (bSSFP) imaging and 3D contrast-enhanced cardiovascular magnetic resonance angiography (CE-CMRA) regarding diagnostic image quality and image artifacts was performed using Likert scales. The scoring data of the two readers are given as absolute frequencies and percentages. Wilcoxon signed rank test was used to intraindividually compare the two sequence techniques. *P* < .05 indicated significant differences. The Intraclass Correlation Coefficient (ICC) was used to assess the interrater agreement. Its 95% confidence interval is given in square brackets

### Artifact scoring at the level of the aortic root graft

Reader 1 found severe or moderate artifacts in 9% of bSSFP scans (0% in CE-CMRA) and scored 91% with minor or no artifacts (100% in CE-CMRA). Reader 2 found severe or moderate artifacts in 13% of bSSFP scans (11% in CE-CMRA) and scored 88% with minor or no artifacts (89% in CE-CMRA). The higher frequency of artifacts observed in bSSFP imaging did not reach the level of significance for both readers (reader 1, *p* = .18; reader 2, *p* = .12). ICC analyses showed superior inter-rater agreement in bSSFP imaging (bSSFP, 0.81; CE-CMRA, 0.65). Detailed results of image artifact ratings are given in Table 1.

### Scoring of aortic dissection

**Fig. 2:**
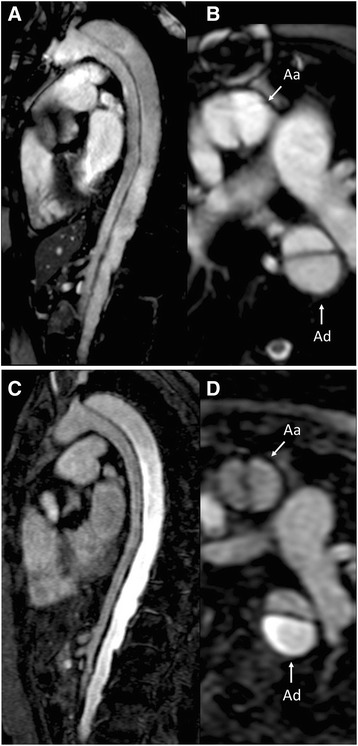
Aortic dissection in bSSFP and CE-CMRA: Residual aortic dissection 18-years after aortic root replacement due to acute type A dissection in a 72 year-old woman with Marfan syndrome. Both non-contrast 2D SSFP in (**a**) parasagittal and (**b**) transversal plane as well as 3D CE-CMRA in (**c**) parasagittal and (**d**) transversal orientation clearly display the dissection membrane visible in the ascending (Aa) and descending aorta (Ad) with high image quality (4 points by both readers). The CE-CMRA provides additional information regarding the perfusion of lumens and shows hyperperfusion of the larger false lumen in the descending aorta in this case

Reader 1 and reader 2 detected all of the 14 dissections with both bSSFP and CE-CMRA, resulting in a sensitivity of 100% for both readers and techniques. There were no false-positive ratings regarding both readers and techniques resulting in a specificity of 100%. Corresponding AUCs of reader 1 and reader 2 were 1.00 (CI: 1.00–1.00) for both techniques. The inter-rater agreement was excellent in both bSSFP (ICC 0.98, CI: 0.97–0.99) and CE-CMRA (ICC 0.99, CI: 0.99–1.00). Figure 2 shows the comparison of bSSFP and CE-CMRA in a patient with known residual type A aortic dissection.

### Aortic diameter measurements and observer agreement

CE-CMRA resulted in statistically significant larger diameters at all aortic levels (bias: 0.5–1.3 mm) when compared to non-contrast bSSFP imaging (all *p* < .05). Pearson’s correlation analyses revealed moderate to strong correlation of diameters obtained by bSSFP imaging and CE-CMRA at all aortic levels (*r* = 0.66–0.96). Detailed results of diameter measurements are provided in Table 2.

**Table 2:** Comparison of aortic diameters as determined by 2D bSSFP and 3D CE-CMRA imaging

2D bSSFP vs. 3D CE-CMRA	Mid Graft	Distal anastomosis	Ascending aorta	Aortic arch	Descending aorta	Diaphragm	Coelic trunk
Mean diameter 2D bSSFP (mm)	32.9	29.1	31.7	27.9	28.0	26.4	25.8
Mean diameter 3D CE-CMRA (mm)	33.5	30.2	32.9	28.9	28.8	26.9	26.7
Mean difference (mm)	−0.5	−1.1	−1.3	−1.0	−0.8	−0.6	−0.9
Limits of agreement (mm)	−5.7 to 4.6	−7.1 to 4.9	−4.9 to 2.4	−7.1 to 5.0	−10.5 to 8.9	−5.2 to 4.0	−5.1 to 3.2
Standard deviation (mm)	2.6	3.0	1.9	3.1	4.9	2.3	2.1
Variance (mm^2^)	7.0	9.3	3.5	9.46	24.4	5.4	4.5
Pearson’s correlation (r)	0.75	0.66	0.89	0.74	0.79	0.96	0.96
*P* value (t-test)	**0.005**	**0.009**	**0.011**	**0.011**	**0.042**	**<0.001**	**<0.001**

Comparison of aortic diameters as determined by 2D bSSFP and 3D CE-CMRA imaging as described by Bland and Altman. Provided measurements are the average of reader 1 and reader 2. Pearson’s correlation coefficient (r) for the different imaging modalities is indicated. Paired t-test was performed for comparison of mean diameters. Significant differences are in bold

The mean intra-observer biases of the diameter measurements ranged between -0.1 mm and 0.6 mm in bSSFP imaging vs. -0.2 mm and 0.7 mm in CE-CMRA without a statistically significant difference (all *p* > .05). Also, there was no statistically significant difference between the variances of both bSSFP imaging and CE-CMRA (all *p* > .05). Detailed results of the intra-observer analyses are given in Table 3.

**Table 3:** Intra-observer variance

	Intra-observer variance						
	Mid-Graft	Distal anastomosis	Ascending aorta	Aortic arch	Descending aorta	Diaphragm	Celiac trunk
	2D SSFP						
Mean difference (mm)	0.22	0.38	0.56	0.14	−0.05	0.29	−0.07
Limits of agreement (mm)	−3.18 to 3.62	−4.39 to 5.14	−3.59 to 4.70	−5.22 to 5.49	−4.21 to 4.10	−6.55 to 7.13	−7.64 to 7.51
Standard deviation (mm)	1.74	2.43	2.11	2.73	2.12	3.49	3.87
Variance (mm^2^)	3.01	5.92	4.47	7.47	4.50	12.17	14.95
ICC	0.95	0.88	0.94	0.89	0.98	0.96	0.94
	3D CE-CMRA						
Mean difference (mm)	0.03	−0.23	0.17	0.58	0.19	0.33	0.73
Limits of agreement (mm)	−3.60 to 3.66	−5.54 to 5.07	−2.55 to 2.88	−3.82 to 4.97	−4.32 to 4.72	−5.31 to 5.98	−6.69 to 8.16
Standard deviation (mm)	1.85	2.71	1.38	2.24	2.31	2.88	3.79
Variance (mm^2^)	3.44	7.34	1.92	5.02	5.31	8.29	14.35
ICC	0.92	0.84	0.97	0.93	0.95	0.96	0.92
*p*-value (t test)	0.491	0.140	0.480	0.367	0.589	0.922	0.869
*p*-value (F test)	0.605	0.398	0.089	0.133	0.530	0.144	0.870

Intra-observer variance of measured aortic diameters as determined by non-contrast 2D bSSFP imaging and 3D CE-CMRA as described by Bland and Altman. Intraclass correlation coefficients (ICC) are given for both sequence types. Paired t-test was performed for comparison of mean differences and F test for comparison of variances

The mean inter-observer bias of the diameter measurements ranged between −3.9 mm and 2.1 mm in bSSFP imaging vs. -3.3 mm and 2.7 mm in CE-CMRA. There was no statistically significant difference between the biases of bSSFP imaging and CE-CMRA (all p > .05), except for the level of the distal anastomosis of the aortic root graft (mean difference, bSSFP 0.1 mm vs. 1.4 mm in CE-CMRA; *p* = .02). There was no statistically significant difference between the variances of both bSSFP imaging and CMRA (all *p* > .05), except for the level of the distal anastomosis of the aortic root graft (bSSFP, 95% limits of agreement, ±4.5 mm vs. CE-CMRA, ±7.9 mm; *p* = .001), the descending aorta (bSSFP, 95% limits of agreement ±6.1 mm vs. CE-CMRA, ±12.0 mm; p = <.001), and the celiac trunk (bSSFP, 95% limits of agreement ±14.3 mm vs. CE-CMRA, ±5.3 mm; *p* = <.001). Detailed results of the inter-observer analyses are given in Table 4.

**Table 4:** Inter-observer variance

	Inter-observer variance						
	Mid-Graft	Distal anastomosis	Ascending aorta	Aortic arch	Descending aorta	Diaphragm	Celiac trunk
	2D SSFP						
Mean difference (mm)	2.08	0.08	1.17	0.10	1.97	1.10	0.79
Limits of agreement (mm)	−4.45 to 8.61	−4.43 to 4.59	−4.87 to 7.20	−5.04 to 5.24	−4.10 to 8.03	−6.34 to 8.55	−8.64 to 10.21
Standard deviation (mm)	3.33	2.30	3.1	2.62	3.10	3.80	4.81
Variance (mm^2^)	11.1	5.29	9.47	6.87	9.59	14.43	23.13
ICC	0.69	0.83	0.86	0.90	0.95	0.89	0.85
	3D CE-CMRA						
Mean difference (mm)	2.34	1.38	1.44	0.41	2.69	1.62	0.95
Limits of agreement (mm)	−4.8 to 9.56	−5.72 to 8.47	−3.54 to 6.43	−5.68 to 6.49	−9.31 to 14.70	−5.39 to 8.62	−4.34 to 6.25
Standard deviation (mm)	3.68	3.62	2.54	3.10	6.13	3.57	2.70
Variance (mm^2^)	13.57	13.11	6.47	9.63	37.53	12.77	7.29
ICC	0.48	0.68	0.84	0.78	0.75	0.87	0.88
*p*-value (t test)	0.543	**0.023**	0.385	0.549	0.350	0.064	0.069
*p*-value (F test)	0.443	**0.001**	0.440	0.220	**<0.001**	0.641	**<0.001**

Inter-observer variance of measured aortic diameters as determined by non-contrast 2D bSSFP imaging and 3D CE-CMRA as described by Bland and Altman. Intraclass correlation coefficients (ICC) are given for both sequence types. Paired t-test was performed for comparison of mean differences and F test for comparison of variances. Significant differences are in bold (significant at *p* < .05)

### Other findings

**Fig. 3:**
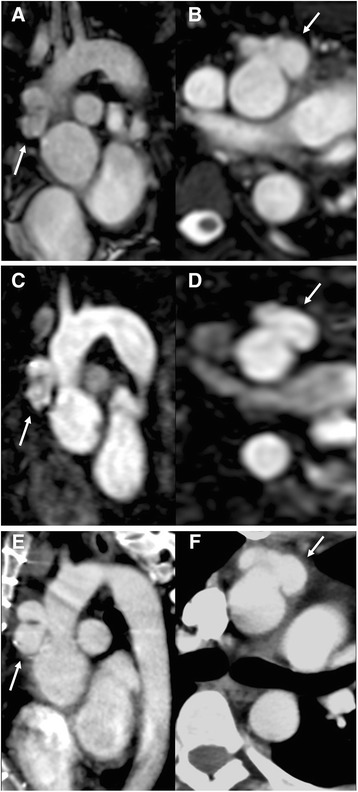
Postoperative suture dehiscence: Postoperative suture dehiscence with consecutive aneurysm at the distal aortic suture line 1-year after David procedure in a 20-year-old man with Marfan syndrome. Both (**a**, **b**) non-contrast 2D bSSFP and (**c**, **d**) 3D CE-CMRA demonstrate the aneurysm (arrows) with high image quality (4 points by both readers). The CMR-based diagnosis was confirmed with (**e**, **f**) computed tomography angiography. The patient underwent immediate surgical revision with re-replacement of the aortic root for treatment of his potentially life-threating complication.

Both readers correctly identified a postoperative aneurysm at the distal aortic suture line of the aortic root graft in both non-contrast bSSFP and CE-CMRA in one of the 64 included patients (1.6%) **(**Fig. 3). Following computed tomography angiography for validation of this finding, the patient underwent surgical revision with re-replacement of the aortic root. No other pathology of the aortic graft region was found in any of the remaining 63 patients (98.4%).

## Discussion

We successfully demonstrated non-contrast bSSFP imaging to provide high image quality, precise and reproducible aortic diameter measurements, and high diagnostic performance in detection of relevant aortic pathologies in Marfan patients after aortic root replacement.

All patients were correctly scored regarding presence of aortic dissections by both readers using both non-contrast bSSFP imaging and CE-CMRA, resulting in 100% sensitivity and specificity for both imaging techniques. Moreover, both readers correctly detected a postoperative aneurysm at the distal aortic suture line with both imaging techniques in a patient after David procedure (Fig. 3). Thus, both bSSFP and CE-CMRA provided comparable diagnostic performance regarding monitoring of aortic dissection and detection of other potentially life-threating complications in postoperative Marfarn patients.

Both observers found no significant differences in overall image quality regarding the two imaging techniques. Former studies in pre-operative patients found superior image quality of bSSFP sequences when compared to CE-CMRA [19, 21]. However, this superior image quality was limited to the aortic root and the ascending aorta. These aortic levels are permanently in motion due to the cardiac cycle and therefore benefit most from ECG-gating, resulting in less blurring of the aortic wall structures in bSSFP images. In contrast, rather motionless distal aortic parts showed comparable image quality in the mentioned pre-operative studies, matching the observations in this report of postoperative Marfarn patients. Moreover, it is likely that the advantage of ECG-gating in bSSFP imaging regarding image quality at the aortic root is mitigated by artifacts caused by the implanted graft. Indeed, results of the subjective scoring of both readers revealed more artifacts at the aortic root graft when using the non-contrast bSSFP sequence at 1.5 Tesla, however, without reaching a statistical significant difference. As bSSFP techniques are prone to off-resonance effects typically manifesting as banding artifacts, more and stronger artifacts caused by surgical material at the stent graft implantation site and the surgical entryway are to be expected [17, 33]. Therefore, unfortunate localization of banding artifacts in bSSFP sequences may require alternative sequences for correct evaluation of the proximal and distal aortic anastomosis in particular cases. As off-resonance artifacts are pronounced at higher field strengths [33, 34], future studies need to address the image quality and artifacts of bSSFP imaging in postoperative Marfarn patients at 3 Tesla.

The diameter measurements revealed a good correlation between bSSFP and CE-CMRA. However, CE-CMRA revealed significantly larger diameters (0.5–1.3 mm) at all measurement points compared to the non-contrast bSSFP sequence. This may be explained by acquisition of the CE-CMRA throughout the cardiac cycle, comprising information from systole and diastole, during which the aortic diameter changes. The resulting blurred aortic wall structures may lead to overestimation of the diameters compared to bSSFP imaging, which was triggered to the diastole as recommended by current guidelines [11]. Previous studies have shown that ECG-gating also improves the image quality of CE-CMRA [35]. However, we do not pursue these ECG-gated contrast-enhanced techniques at our institution, as we strive to avoid the use of contrast material for repeated life-long imaging of Marfarn patients.

Both, bSFFP imaging and CE-CMRA revealed excellent intra-rater agreement of diameter measurements at all aortic levels, serving as marker for high reproducibility. Regarding inter-rater agreement, a significant higher variability was found for CE-CMRA than for bSSFP imaging at the distal graft anastomosis. This difference is likely explained by blurring of aortic wall structures due to cardiac motion in CE-CMRA, making reliable and exact measurements challenging.

The results acknowledge bSSFP imaging as an equivalent imaging technique when compared to CE-CMRA regarding aortic monitoring in postoperative Marfan patients. The reported observations have important clinical implications: At our institution, we stopped application of intravenous contrast for routine MR imaging of the thoracic aorta at 1.5 Tesla in asymptomatic postoperative Marfarn patients without known aortic dissection and perform bSSFP imaging only. Only if aortic dissection is known to be present or newly found on non-contrast SSFP, we continue to acquire a CE-CMRA to assess contrast dynamics within the true and false lumens of the dissected aorta (Fig. 3) and for improved three-dimensional visualization of the exact extent of the dissection e.g. within the supraaortic branches.

Beside the patients with aortic dissection, only one other aortic complication after root surgery was observed in the study population (patient with aneurysm at the distal aortic suture line). This limits the validity of the sequence comparison regarding diagnostics of aortic pathologies other than aortic dissection. However, both readers correctly identified this severe complication with both imaging techniques. The reoperation rate over 10 years after aortic root replacement due to postoperative complications has been indicated with 6% in Marfan patients, matching the observed single complication in this study population [36].

Some technical parameters of the used 2D bSFFP and the 3D CE-CMRA sequences did not fully match. This is the case, because a product sequence was used for bSSFP imaging without further adjusting resolution and volumetric coverage to the preset of the CE-CMRA sequence used in our institution, which was also a product sequence. It may be regarded as another study limitation that measurements were performed in para-sagittal planes (along the flow axis of the aorta) without using secondary multiplanar reformations. However, as the orientation of both 2D bSSFP and 3D CE-CMRA were identical, we believe that secondary reformations would not have provided significantly different results. In clinical practice, particularly when the aortic diameters reach threshold values, secondary reformations can be performed to assess the maximal diameter of the aorta in different orientations.

Last, it should be recognized that although the readers performed individual reading of anonymized and randomized images acquired with bSSFP and CE-CMRA sequences, a true blinding was not possible since it is obvious to a reader whether a contrast agent was administered.

## Conclusion

In summary, 2D bSSFP imaging and 3D CE-CMRA resulted in equivalent image quality, thereby providing comparable performance regarding detection of aortic dissection and aneurysm as well as reliable aortic diameter assessment in Marfan patients after aortic root replacement. The results acknowledge non-contrast bSSFP imaging of the thoracic aorta as an appropriate alternative for serial monitoring of patients with Marfan syndrome after aortic root surgery. Renouncement of intravenous gadolinium contrast avoids adverse effects and facilitates patient management. Only when aortic dissection is known to be present or newly detected on unenhanced bSSFP images, additional acquisition of a CE-CMRA is recommended.

## Abbreviations

AUC: Area under the curve
bSSFP: balanced steady state with free precision
CE-CMRA: Contrast-enhanced CMRA
CI: Confidence interval
CMRA: Cardiovascular magnetic resonance angiography
ECG: Electrocardiogram
ROC: Receiver operating characteristic
TE: Echo time
TR: Repetition time
